# Wardell on Enteric Fever

**Published:** 1861-09

**Authors:** 


### WARDELL ON ENTERIC FEVER.

Reprinted from *London Lancet* per Braithwaite.

[Of course our readers are aware that, by those who have had most opportunity of judging, fevers are divided into typhus, enteric or typhoid, relapsing, and febricula. The two first are the principal and of most importance. Dr. Wardell published some papers on this subject fourteen years ago, founded on the observation of 1200 cases of fever in the hospitals of Edinburgh. At that time he believed enteric and typhus fever to be the same.]

Enteric fever begins more insidiously than typhus, and much more so than the relapsing. In the latter, the attack is sometimes quite sudden; a person may go to bed comparatively well, and in the morning be seized with shivering, headache, pains in the limbs, and other symptoms. There is less dulness and stupidness in the enteric than in typhus, in which the great nervous centres seem prone to be more readily impressed, as if the poison at once affected animal life. It is quite true, however, that all forms of continued fever in the symptomatology of accession present much in common, and, under an experienced eye, it is often difficult during the first few days, or even until the rash appears, to arrive at a decided diagnosis. In typhus, the muscular system evinces great prostration, not only at the first, but throughout the disease; as a rule, the patient lies helplessly on his back.

Dr. Jenner has noticed that, in the enteric type, patients have a greater tendency to "get out of bed" than in typhus; the delirium is of a more vivacious kind; they are less easily restrained. In many cases the delirium does not come on until the second or third week; when it does in the first week, it is to be looked upon unfavorably. In typhus it supervenes before the end of the first week, when it increases, and in fatal cases ends in coma. In relapsing fever, there is comparatively little delirium and head complication. On reference to my own account of that disease, in one table giving particulars of 450 cases, leeches were applied to the head, on the average, to 1 in 6-62; in another set of 80 cases, it was a predominating symptom in the small proportion of 1 in 11-42. According to Louis, the brain and its membranes, of those dying of the enteric, rarely present any such marked appearance as might be deemed of potential consequence entering into the causation of death. To those who have had considerable experience in morbid anatomy, it is well known that the encephalic mass gives little explanation of the essential nature of coutinued fevers. A small amount of subarachnoid and ventricular effusion, a pinkish flush of the cortical substance, or it may be a few puncta in the centrum ovale, constitute often the entirety of morbid phenomena, and I have repeatedly examined this organ when it evinced no lesion. Even coma may not be traced after death. There is no doubt that the views enunciated by the late Dr. Clutterbuck, and those professing similar opinions—regarding fever to consist of inflammation of the brain—led to much mischief in practice, and that a more correct pathology (which is, it cannot be denied, an approach to the once widely-received and then discarded humoral doctrine) has been succeeded by a more successful mode of treatment—viz., that treatment, which, as far as possible, dispenses with depletive measures, which is more expectant and conservative in its aims, and which, by having a just relation to a set of morbid actions not primarily located in any single organ or separate tissue, but pervading the entire organism, opposes asthenia. The late Dr. Alison, whose clinical assistant I was, and whose practice I carefully observed, used to dwell with much emphasis upon the Cullenian maxim of "averting the tendency to death"; and although he did not carry the wine and brandy remedy to the extreme, which, by some, has latterly been advocated, yet, he had long seen, with much sagacity, that acute diseases, and more especially fevers, had, from some subtle and inexplicable cause become less sthenic in their nature, demanding remedies, which, in previous years, were accounted inapplicable or decidedly prejudicial.

Those organs and surfaces which are of an eliminative, a depurative, or defecating character—those emunctories and outlets whereby the products of organic waste and effete matter are carried off, are precisely those which are most liable to become affected in the progress of fever. That poison, which, by a most rapid multiple, has so vastly increased as to contaminate the whole of the circulating fluid, in obedience to certain vital laws inherent in the organism, by such channels becomes expelled, hence, according to the power of such agent, the excess or defect of function of such organs and surfaces; and thus it is that the skin, the mucous membranes, and the parenchyma of secernant viscera more especiallv manifest the effects of the specific poison. Ancient theory and every-day facts convince us of those conservative qualities which the system possesses, whereby it essays to eject what it cannot assimilate, and to get rid of such noxious matter as it may have contracted. In the exanthemata, the skin and the mucuous membranes of the air-passages are the chief seats of its determinations; in enteric fever, the pustulation is in the digestive tube, mainly in the ileum; in typhus, the mulberry rash evinces an effort made at the superficies to throw off the poison; and in relapsing fever, the powerful diaphoresis which so frequently at once resolves the fever, inculcates the same doctrine. It now being conceded that the three eruptions peculiar to the respective types of continued fever, are as pathognomonic of those varieties as are the eruptions in the exanthemata, properly so called, it would really seem that it is merely an arbitrary distinction on the part of systematizers and compilers of nosology, whereby they are differently arranged. They all have many features in common; they all originate from peculiar morbid poisons, requiring a greater or less period of incubation; they all pass through a certain train of febrile phenomena; and in all the great centres of animal and organic life are in varied degree affected. Again, there are many points of resemblance in the complications which arise, in the demand of treatment, in the sequelæ, and in the phenomena of the fatal issue. If we were to make a hasty comparison between small-pox and spotted typhus, how many characteristics they manifest of a like nature, still unquestionably caused by poisons, with which like only produces like. Both originate from peculiar poisons, are contagious, require a certain period for development, are diseases in which the prime agent specially operates upon and multiplies in the blood; in one the eruption may be looked for on the third, in the other on the sixth day; in both are all the pyrexial symptoms, as a quick circulation, high temperature, furred tongue, anorexia, diminished secretions, and a great impress is made on the nervous system; in each organic complications of the inflammatory kind may arise; there is delirium and often coma. In the one form, death mostly takes place on or about the eleventh day; in the other, rarely till after the twentieth; and both may have sequelæ attacking the same organs and tissues. And similar comparisons might be drawn between any two types taken from continued fevers and the exanthems. Although human reasoning cannot say why it is that in one variety the pustulation is in the intestines, in another in the skin, yet accumulated observations have long shown, that between the skin and the mucous membrane of the digestive tract there is a peculiar sympathy, and that between the enteric and variolous phenomena, many of the fundamental symptoms exemplify no slight or casual features of resemblance.

Dr. Kennedy, of Dublin, in a paper read a few months ago, before the Medical and Chirurgical Society, propounds the doctrine that typhus and typhoid (enteric) are mere varieties originating from the same poison. Dr. Jenner's facts are opposed to this opinion. Of relapsing and typhus I can speak with much certainty. Sixteen years ago I maintained from very elaborate data their distinct essence, and such doctrine still holds good. In more than 1200 cases, I never saw typhus and relapsing blended. The infection caught from one fever never produced the other. Like always produced like in a multitude of instances. I have given in my papers thirty-two cases, in which the two forms succeeded each other within a short space of time. Seventeen out of the thirty-two who had passed through the relapsing, contracted typhus during convalescence, or within the brief period of three months. The proofs of the non-identity of their essential cause were as clear as the common-sense proofs we have, and as practice ever tells us of the non-identity of small-pox and scarlet fever. If typhus differs from the relapsing, why may it not differ from the enteric?

